# RETRACTION: Comparing Surgical Site Wound Infection After Laparoscopic and Open Radical Cystectomies in Patients with Bladder Cancer

**DOI:** 10.1111/iwj.70263

**Published:** 2025-02-25

## Abstract

**RETRACTION:** LuoH.‐M. and XiongY.‐J. , “Comparing Surgical Site Wound Infection After Laparoscopic and Open Radical Cystectomies in Patients with Bladder Cancer,” International Wound Journal 21, no. 4 (2024): e14718, 10.1111/iwj.14718.38571455 PMC10993015

The above article, published online on 04 April 2024, in Wiley Online Library (http://onlinelibrary.wiley.com/), has been retracted by agreement between the journal Editor in Chief, Professor Keith Harding; and John Wiley & Sons Ltd. Following an investigation by the publisher, all parties have concluded that this article was accepted solely on the basis of a compromised peer review process. The editors have therefore decided to retract the article. The authors did not respond to our notice regarding the retraction.

**RETRACTION:** H.‐M. Luo and Y.‐J. Xiong , “Comparing Surgical Site Wound Infection After Laparoscopic and Open Radical Cystectomies in Patients with Bladder Cancer,” International Wound Journal 21, no. 4 (2024): e14718, 10.1111/iwj.14718.38571455 PMC10993015

The above article, published online on 04 April 2024, in Wiley Online Library (http://onlinelibrary.wiley.com/), has been retracted by agreement between the journal Editor in Chief, Professor Keith Harding; and John Wiley & Sons Ltd. Following an investigation by the publisher, all parties have concluded that this article was accepted solely on the basis of a compromised peer review process. The editors have therefore decided to retract the article. The authors did not respond to our notice regarding the retraction.

